# Treatment of human intestinal cryptosporidiosis: A review of published clinical trials

**DOI:** 10.1016/j.ijpddr.2021.09.001

**Published:** 2021-09-21

**Authors:** Ajib Diptyanusa, Ika Puspa Sari

**Affiliations:** a Department of Parasitology, Faculty of Medicine, Public Health and Nursing, Universitas Gadjah Mada, Indonesia; b Study Program of Medical Specialist in Clinical Parasitology, Faculty of Medicine, University of Indonesia, Indonesia; c Department of Parasitology, Faculty of Medicine, University of Indonesia, Indonesia

**Keywords:** *Cryptosporidium*, Cryptosporidiosis, Treatment, Drug, Clinical trial, Clearance

## Abstract

The global burden of diarrhea caused by *Cryptosporidium* parasite is underestimated. In immunocompromised hosts, chronic and severe presentation of intestinal cryptosporidiosis can result in long-term morbidity and high illness costs. The evidence of effective treatments for cryptosporidiosis has been lacking. We reviewed the published clinical trials to bring forward the feasible therapeutic options of human cryptosporidiosis in various populations and settings according to clinical improvement and parasite clearance rates. A total of 42 studies consisting of the use of nitazoxanide, paromomycin, macrolides, somatostatin analogues, letrazuril, albendazole, rifaximin, miltefosine, clofazimine, and colostrum were included in the review. The trials were mostly conducted in small number of individuals infected with human immunodeficiency virus (HIV), and there is inadequate data of controlled trials to suggest the use of these treatment modalities. Nitazoxanide was reported to be highly efficacious only in immunocompetent hosts and was found to be superior to paromomycin in the same group of patients. Macrolides showed no effective results in both clinical and parasitological improvement. Human bovine colostrum should possibly be administered as one of complementary therapeutic modalities along with other antimicrobials to reach optimal parasite eradication. Other trials of therapeutic modalities were terminated due to futility. Currently, available data is intended to aid the development of strategies for improving access to treatments in different clinical settings, as well as to help guide further studies on treatments of human intestinal cryptosporidiosis.

## Graphical abstract

**Figure:**
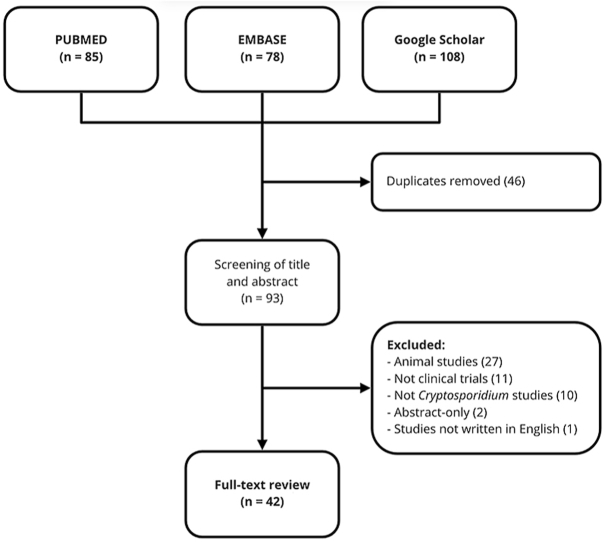

## 1 Introduction

*Cryptosporidium* belongs to the Phylum Apicomplexa, which infects a wide range of vertebrate hosts, including humans (Gerace et al., 2019). This intracellular protozoan parasite is generally transmitted through ingestion of food or water contaminated with its oocysts (Samie et al., 2015). *Cryptosporidium* infects the gastrointestinal epithelium, causing diarrhea that is mostly mild yet sometimes debilitating in certain groups of individuals (McCann, 2019). The first human cases of cryptosporidiosis were noticed in 1976, and the parasite was then recognized as an opportunistic pathogenic parasite associated with diarrhea in immunocompromised individuals (Meisel et al., 1976; Nime et al., 1976). Along with the increasing incidence of human immunodeficiency virus (HIV) infection worldwide in the early 1980s, *Cryptosporidium* became more widely known as acquired immunodeficiency syndrome (AIDS)-defining disease (Ahmadpour et al., 2020; Ashigbie et al., 2021).

Outbreaks of human cryptosporidiosis are not only reported in developing countries, but also in industrialized countries (Mac Kenzie et al., 1994; Shirley et al., 2012; Ursini et al., 2020), mainly attributable to its low infective dose, the presence of wide range of animal reservoirs, and its ability to withstand chlorination (Adeyemo et al., 2019; Bouzid et al., 2013; DuPont et al., 1995). While the disease may be asymptomatic in immunocompetent human hosts, cryptosporidiosis is more likely to be chronic and more severe in immunocompromised individuals, and may result in high illness costs and long-term manifestations such as post-infectious inflammatory bowel syndrome in both immunocompetent and immunocompromised groups (Jadallah et al., 2017; Monge et al., 2019; O'Connor R et al., 2011). *Cryptosporidium* has also been recognized as the leading cause of severe and life-threatening diarrhea in children, particularly in developing countries (Khalil et al., 2018). Despite its long history of recognition over the past decades, the evidence of effective antiparasitic treatments for cryptosporidiosis has been lacking. Even with the initiation of effective antiretroviral (ARV) therapy to restore the immune function of immunocompromised hosts, chronic diarrhea caused by cryptosporidiosis is associated with high mortality (Dillingham et al., 2009). Nitazoxanide is currently the only drug approved by the FDA to treat cryptosporidiosis (Checkley et al., 2015), yet it is unavailable in many developing countries where the disease burden is prominent. Diverse therapeutic modalities have been studied in case reports, case series and clinical trials with different results. However, there is inadequate data to recommend the use of some of the studied treatment approaches. The current review aimed to address the feasible therapeutic options of human cryptosporidiosis in various populations and settings according to published clinical trials.

## 2 Methods

**Fig. 1:**
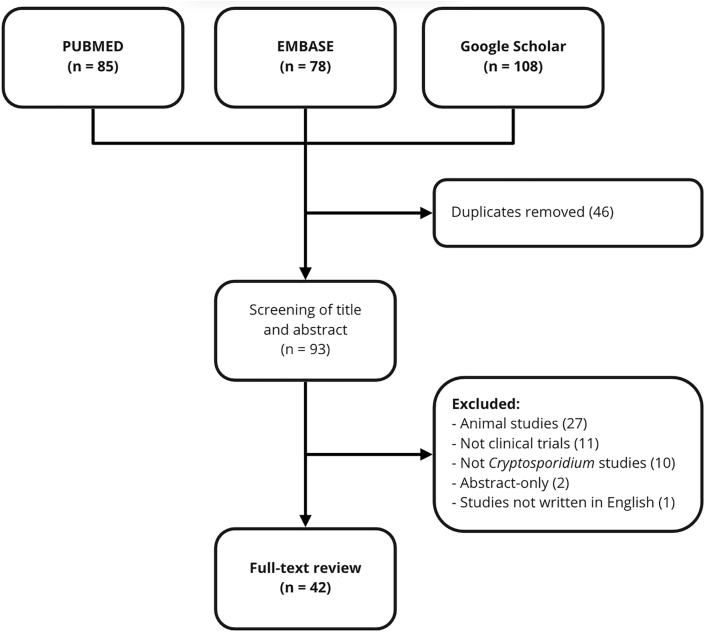
Literature search flow.

Literature searching was performed using three online databases, including PubMed (MEDLINE), EMBASE, and Google Scholar. Inclusion criteria were published results of clinical trials written in English and related to the treatment of human intestinal cryptosporidiosis. Truncations and Boolean terms were utilized to facilitate literature searching. Combinations of the following search terms were used for article explorations: *Cryptosporidium, treatment, drug, trial, random, nitazoxanide, paromomycin, azithromycin, spiramycin, roxithromycin, somatostatin, letrazuril, albendazole, rifaximin, miltefosine, clofazimine,* and *colostrum*. The search yielded a total of 271 articles. After removing duplicates and applying inclusion and exclusion criteria, 42 studies were included in this review (Fig. 1).

## 3 Results

Overall, included studies were published from late 1980s through 2020, the majority of which were published in the 1990s (Table 1). The trials were mostly conducted in individuals living with HIV of various stages or other comorbid leading to immunosuppression. Open-label trials are defined as clinical trials, in which both study participants and researcher were aware of the treatment allocations in the study. In general, primary endpoints for these studies include clinical improvement that was defined as either abatement of diarrhea or reduced stool frequency or volume, and parasite clearance that was defined as either complete eradication or reduction of *Cryptosporidium* oocysts.

**Table 1:** Summary of reviewed studies on treatments of human intestinal cryptosporidiosis.

Treatment modalities	Publication year	Country	Study design	Population	Size	Ref
Nitazoxanide	1997	Mali	Open-label clinical trial	>12 yo with advanced HIV	14	Doumbo et al. (1997)
	1998	Mexico	RCT	18-65 yo with HIV	54	Rossignol et al. (1998)
	2001	Egypt	RCT	1-65 yo healthy individuals	99	Rossignol et al. (2001)
	2002	Zambia	RCT	1-8 yo with HIV or healthy individuals	96	Amadi et al. (2002)
	2006	USA	Open-label clinical trial	≥3 yo with HIV	357	Rossignol (2006)
	2006	Egypt	RCT	≥12 yo healthy individuals	86	Rossignol et al. (2006)
	2009	Zambia	RCT	1-11 yo with HIV	52	Amadi et al. (2009)
	2013	Egypt	Open-label clinical trial	6 mo to 10 yo with malnutrition	135	Hussien et al. (2013)
	2015	Pakistan	Open-label clinical trial	>16 yo healthy individuals	58	Ali and Kumar (2015)
	2016	Egypt	RCT	1-12 yo immune-competent and -compromised individuals	120	Abaza et al. (2016)
Paromomycin	1993	USA	Open-label clinical trial	30-49 yo with HIV	7	Fichtenbaum et al. (1993)
	1994	France	Open-label clinical trial	25-62 yo with HIV	24	Bissuel et al. (1994)
	1994	USA	RCT	25-38 yo with HIV (CD4 <100 cells/uL)	10	White et al. (1994)
	1996	USA	Open-label clinical trial	Adults with HIV (CD4 ≤200 cells/uL)	44	Flanigan et al. (1996)
	1998	USA	Open-label clinical trial	23-44 yo with HIV (CD4 <100 cells/uL)	11	Smith et al. (1998)
	2000	USA	RCT	≥13 yo with HIV (CD4 ≤150 cells/uL)	31	Hewitt et al. (2000)
	2013	Egypt	Open-label clinical trial	6 mo to 10 yo with malnutrition	135	Hussien et al. (2013)
Macrolides	1987	Canada	N of 1 trial	24 yo with HIV	1	(Woolf et al., 1987)
	1988	London	Open-label clinical trial	All age with HIV	15	Connolly et al. (1988)
	1989	South Africa	RCT	<1 yo immune-compromised infants	39	Wittenberg et al. (1989)
	1998	Brazil	Open-label clinical trial	23-49 yo with HIV	24	Sprinz et al. (1998)
	1998	Brazil	Open-label clinical trial	Adults with HIV	22	Uip et al. (1998)
	1998	Italy	Open-label clinical trial	30-47 yo with HIV (CD4 <200 cells/uL)	13	Dionisio et al. (1998)
	2002	India	RCT	22-63 yo with HIV	41	Kadappu et al. (2002)
	2015	China	RCT	>20 yo drug users	151	Huang et al. (2015)
Bovine colostrum	1990	Australia	RCT	Individuals with HIV	5	Nord et al. (1990)
	1990	USA	RCT	31-46 yo with HIV	14	McMeeking et al. (1990)
	1992	Germany	Open-label clinical trial	1-54 yo with HIV and other immune deficiencies	7	Rump et al. (1992)
	1993	Germany	Open-label clinical trial	26-58 yo with HIV (CD4 <100 cells/uL)	7	Plettenberg et al. (1993)
	1996	USA	Open-label clinical trial	≥18 yo with HIV (CD4 <200 cells/uL)	20	Greenberg and Cello (1996)
	2005	Nigeria	Open-label clinical trial	≥18 yo with HIV (CD4 <200 cells/uL)	20	Florén et al. (2006)
Somatostatin analogue	1991	USA	Open-label clinical trial	18-60 yo with HIV	15	Cello et al. (1991)
	1991	Spain	Open-label clinical trial	22-48 yo with HIV	18	Romeu et al. (1991)
	1992	Italy	Open-label clinical trial	37-70 yo with HIV (CD4 <100 cells/uL)	4	Liberti et al. (1992)
	1992	France	Open-label clinical trial	≥18 yo with HIV	21	Girard et al. (1992)
	2009	Italy	Open-label clinical trial	21-60 yo with HIV (CD4 <100 cells/uL)	13	Moroni et al. (1993)
Letrazuril	1994	USA	Open-label clinical trial	21-59 yo with HIV (CD4 <100 cells/uL)	14	Harris et al. (1994)
	1995	Canada	Open-label clinical trial	27-57 yo with HIV (CD4 <150 cells/uL)	35	Loeb et al. (1995)
Rifaximin	1999	Italy	Open-label clinical trial	12-54 yo with HIV	10	Amenta et al. (1999)
Albendazole	2002	Zambia	Open-label clinical trial	≥18 yo with HIV (CD4 <200 cells/uL)	4	Zulu et al. (2002)
Miltefosine	2011	Zambia	Open-label clinical trial	≥18 yo with HIV and malnutrition	7	Sinkala et al. (2011)
Clofazimine	2020	Malawi	RCT	18-65 yo with HIV (severe immunosuppression)	20	Tam et al. (2020)
Probiotics	2014	India	RCT	6 months to 5 yo healthy children	42	Sindhu et al. (2014)

RCT: randomized clinical trial.

### 3.1 Nitazoxanide

Among the drugs proposed for the treatment of cryptosporidiosis, nitazoxanide is the most frequently studied. Various doses have been assigned in the studies as shown in Table 2. An open-label clinical trial in individuals living with HIV using the dose of 500 mg administered twice daily for 7 days resulted in 64% clinical improvement and 57% parasite clearance (Doumbo et al., 1997). A similar dose with a shorter treatment duration gave a better outcome in both clinical parameters and oocyst excretion (Rossignol et al., 2006). However, the study was conducted in healthy individuals with the potential for a positive impact on the overall treatment outcome. Better treatment responses after the use of nitazoxanide were also reported in immunocompetent hosts rather than those in immunocompromised hosts (Abaza et al., 2016; Amadi et al., 2002). Diarrhea episodes resolved within 3–5 days in immunocompetent group, and within 21–28 days in immunocompromised group (Abaza et al., 2016). Similarly, another study also showed larger proportion of HIV-seronegative children had the diarrhea episodes resolved within 7 days after initiation of nitazoxanide (Amadi et al., 2002). Additionally, other studies performed in healthy individuals revealed clinical improvement in terms of abatement of diarrhea up to 80–100% (Ali and Kumar, 2015; Rossignol et al., 2001) and eradicating parasite in 67% of the patients (Rossignol et al., 2001). Higher dose of nitazoxanide and longer duration of treatment did not substantiate higher cure rate in some studies (Amadi et al., 2009; Rossignol, 2006; Rossignol et al., 1998). Frequently reported side effects include gastrointestinal upset, fatigue, drowsiness, and headache (Ali and Kumar, 2015; Amadi et al., 2009; Rossignol, 2006; Rossignol et al., 1998, 2001, 2006). Scleral and urine discoloration have also been reported although uncommon (Amadi et al., 2009; Rossignol, 2006; Rossignol et al., 2006). According to the available published data regarding efficacy of nitazoxanide on human cryptosporidiosis in controlled trials, the benefit of nitazoxanide may be limited only to immunocompetent individuals. This is showed by shorter duration of diarrhea and better parasite clearance of healthy individuals compared to that of immunocompromised group (Abaza et al., 2016; Amadi et al., 2002). However, nitazoxanide did not demonstrate superiority over placebo in HIV individuals (Amadi et al., 2009; Rossignol et al., 1998). Nitazoxanide acts by inhibiting the pyruvate:ferredoxin oxidoreductase (PFOR), an enzyme essential for the metabolism of certain anaerobic bacteria and parasites (Hoffman et al., 2007). The PFOR in *Cryptosporidium* has an atypical arrangement in which the enzyme contains a C-terminal cytochrome P450 protein, hence Nitazoxanide action via PFOR remains debatable (Bartelt et al., 2018). Furthermore, observed better efficacy of nitazoxanide in immunocompetent patients, but less so or not at all in immunodeficient individuals might have suggested that host immunity contributed to drug effectiveness (Miyamoto and Eckmann, 2015).

**Table 2:** Studies on the use of nitazoxanide.

Study	Population	Size	Disease severity	Regimen	Comparison	Follow up period	Clinical improvement^a^	Parasite clearance^b^
Doumbo et al. (1997)	>12 yo with advanced HIV	14	All intensity	500 mg BID for 7 days	None	Days 7 and 14	64% (9/14)	57% (8/14)
Ali and Kumar (2015)	>16 yo healthy individuals	58	All intensity	500 mg BID for 7 days	None	Day 14	100%	N/A
Rossignol (2006)	≥3 yo with HIV	357	All intensity	500–1500 mg BID for maximum 30 days	None	Weeks 1, 2, 4	59% (209/357)	33% (116/357)
Rossignol et al. (2001)	1-65 yo healthy individuals	99	All intensity	100 mg (1–3 yo), or 200 mg (4–11 yo), or 500 mg (adults) BID for 3 days	Placebo	Day 7	80% (39/49) vs. 41% (20/49)^a^	67% (33/49) vs. 22% (11/50)^b^
Amadi et al. (2002)	1-8 yo with HIV or healthy individuals	96	All intensity	100 mg BID for 3 days	Placebo	Day 7	*HIV*: 8% (2/25) vs. 25% (6/24)^b^ *Non-HIV*: 56% (14/25) vs. 23% (5/22)	*HIV*: 16% (4/25) vs. 21% (5/24)^b^ *Non-HIV*: 52% (13/25) vs. 14% (3/22)
Abaza et al. (2016)	1-12 yo immunocompromised and immunocompetent individuals	120	All intensity	100 mg (<3yo), or 200 mg (>3 yo) BID for 3–4 days (ICT), or 7–28 days (ICZ)	Placebo	At the end of treatment	*Immunocompromised*: 63% (19/30) vs. 10% (3/30)^a^ *Immunocompetent*: 97% (29/30) vs. 53% (16/30)^a^	*Immunocompromised*: 53% (16/30) vs. 23 (7/30)^a^ *Immunocompetent*: 93% (28/30) vs. 43% (13/30)^a^
Rossignol et al. (2006)	≥12 yo healthy individuals	86	All intensity	500 mg BID for 3 days	Placebo	Days 7 and 10	92% (54/59) vs. 41% (11/27)^a^	92% (54/59) vs. 37% (10/27)^a^
Amadi et al. (2009)	1-11 yo with HIV	52	All intensity	200 mg (1–3 yo), or 400 mg (4–11 yo) BID for 28 days	Placebo	Every other day until parasite clearance	42% (11/26) vs. 35% (8/26)^b^	27% (7/26) vs. 35% (8/26)^b^
Rossignol et al. (1998)	18-65 yo with HIV	54	All intensity	500 mg BID for 14 days	Nitazoxanide 1 g BID for 14 days and placebo	Days 7, 15, 22 and 29	63% (12/19) vs. 60% (9/15) vs. 50% (10/20)^a^	63% (12/19) vs. 67% (10/15) vs. 25% (5/20)^a^
Hussien et al. (2013)	6 mo to 10 yo with malnutrition	135	All intensity	100 mg (<3yo), or 200 mg (>3 yo) BID for 3 days	Paromomycin 25 mg/kg/d for 14 days	At the end of treatment	87% (39/45) vs. 69% (31/45)^a^	87% (39/45) vs. 69% (31/45)^a^

^a^*P* < 0.05.

^b^Not statistically significant.

NS: not statistically significant.

a Either abatement of diarrhea or reduced stool frequency or volume.

b Either complete eradication or reduction of oocyst number.

### 3.2 Paromomycin

The efficacy of a non-absorbable aminoglycoside called paromomycin has also been studied in several clinical trials in the treatment of human cryptosporidiosis. Paromomycin inhibits protein synthesis by binding to the prokaryotic ribosomes of *Cryptosporidium*, hence preventing the infection (Marshall and Flanigan, 1992). In the published studies, all were conducted in individuals with immunocompromised status (Table 3). Some of the studies conducted in HIV-infected individuals with CD4 levels of ≤200 cells/uL resulted in clinical improvement of less than 50% and a low degree of parasite clearance (Flanigan et al., 1996; Hewitt et al., 2000; Smith et al., 1998; White et al., 1994). The 500 mg dose administered 3–4 times daily for 14–21 days did not seem to give a significantly better outcome in this population compared to placebo (Hewitt et al., 2000; White et al., 1994). Higher dose of paromomycin and the addition of azithromycin for longer treatment duration also yielded sub-optimal results (Smith et al., 1998). Nevertheless, there were studies that reported 92–100% clinical improvement along with 75–92% parasite eradication rate after the administration of paromomycin (Bissuel et al., 1994; Fichtenbaum et al., 1993). However, these research did not give detailed characterization on the severity of patients’ immunocompromised status and were conducted in a relatively small population. Another randomized controlled trial (RCT) conducted in children reported inferior cure rate of paromomycin when compared to nitazoxanide (69%–87%) (Hussien et al., 2013). However, the latter study was conducted in immunocompetent children. Other than gastrointestinal upset, an alarming side effect of paromomycin was elevated alkaline phosphatases (Hewitt et al., 2000). Reported clinical trials on the use of paromomycin as the treatment of human cryptosporidiosis remain lacking, possibly due to the consistently low efficacy despite extended therapeutic regimen.

**Table 3:** Studies on the use of paromomycin.

Study	Population	Size	Disease severity	Regimen	Comparison	Follow up period	Clinical improvement^a^	Parasite clearance^b^
Fichtenbaum et al. (1993)	30-49 yo with HIV	7	All intensity	500 mg QID for 10–14 days	None	Weekly up to 6 months	100%	75% (3/4)
Bissuel et al. (1994)	25-62 yo with HIV	24	All intensity	1 g BID for 4 weeks, 500 mg BID for maintenance	None	N/A, 3 stool samples	92% (21/24)	92% (21/24)
Flanigan et al. (1996)	Adults with HIV (CD4 ≤200 cells/uL)	44	All intensity	500 mg QID for 4 weeks	None	Weeks 2 and 4	48% (21/44)	46% (12/26)
Smith et al. (1998)	23-44 yo with HIV (CD4 <100 cells/uL)	11	Chronic cryptosporidiosis	1 g paromomycin BID for 8 weeks plus 600 mg azithromycin OD for 8 weeks	None	Weeks 2, 4 and 12	27% (3/11)	60% (6/10)
White et al. (1994)	25-38 yo with HIV (CD4 <100 cells/uL)	10	Chronic cryptosporidiosis	500 mg TID or QID for 14 days	Placebo	Weekly during therapy	50% (3/6) vs. 0	67% (4/6) vs. 0
Hewitt et al. (2000)	≥13 yo with HIV (CD4 ≤150 cells/uL)	31	All intensity	500 mg QID for 21 days	Placebo	Weeks 1, 3, 4, 6, and 9	47% (8/17) vs. 36% (5/14)^b^	35% (6/17) vs. 29% (4/14)^b^
Hussien et al. (2013)	6 mo to 10 yo with malnutrition	135	All intensity	25 mg/kg/d For 14 days	Nitazoxanide 100–200 mg BID for 3 days	At the end of treatment	69% (31/45) vs. 87% (39/45)^a^	69% (31/45) vs. 87% (39/45)^a^

^a^*P* < 0.05.

^b^Not statistically significant.

NS: not statistically significant.

a Either abatement of diarrhea or reduced stool frequency or volume.

b Either complete eradication or reduction of oocyst number.

### 3.3 Macrolides

Table 4 shows the use of macrolides in human cryptosporidiosis included in the current review. The earliest trials on the treatment of cryptosporidiosis include the use of macrolide antibiotics in both *in vitro* and *in vivo* studies. Azithromycin is one of the macrolide antibiotics widely used in these studies, particularly after being found to inhibit the growth of *Cryptosporidium* in culture medium and in immunosuppressed rats (Giacometti et al., 1996; Rehg, 1991). Two studies conducted in HIV-infected adults showed 67%–100% clinical improvement with doses ranged from 500 mg to 1.5 g (Dionisio et al., 1998; Kadappu et al., 2002). A larger dose did not seem to contribute much in the abatement of diarrhea episodes and parasite clearance in these studies. In contrast, longer treatment duration using 500 mg dose over 14 days yielded better oocyst reduction up to 83% (Dionisio et al., 1998; Kadappu et al., 2002). Another case report also showed highly efficacious results in children with cancer using the dose of 10 mg/kg on day 1 and 5 mg/kg on days 2 through 10 (Hicks et al., 1996).

**Table 4:** Studies on the use of macrolides.

Study	Population	Size	Disease severity	Drug(s)	Regimen	Comparison	Follow up period	Clinical improvement^a^	Parasite clearance^b^
Huang et al. (2015)	>20 yo drug users	151	Asymptomatic	Acetylspiramycin plus Garlicin	200 mg plus 40 mg QID for 7 days	Placebo	Day 7	N/A	92% (35/38) vs. 62% (21/34)^a^
Dionisio et al. (1998)	30-47 yo with HIV (CD4 <200 cells/uL)	13	Chronic and relapsed cryptosporidiosis	Azithromycin	500 mg OD for 30–40 days	Azithromycin 1 g OD for 21–50 days and 1.5 g OD for 20 days	Monthly	83% (5/6) vs. 67% (2/3) vs. 75% (3/4)	83% (5/6) vs. 67% (2/3) vs. 75% (3/4)
Kadappu et al. (2002)	22-63 yo with HIV	41	All intensity	Azithromycin	500 mg OD for 5 days	Azithromycin 500 mg OD for 7 days and for 14 days	Days 5, 7, 14	100% in all treatment arms	0 vs. 0 vs. 38% (5/13)
Connolly et al. (1988)	All age with HIV	15	N/A	Erythromycin	500 mg QID for 7 days	Spiramycin 500 mg QID for 7 days	Day 14	100% in all treatment arms	0 vs. 0
Sprinz et al. (1998)	23-49 yo with HIV	24	Chronic cryptosporidiosis	Roxithromycin	300 mg BID for 4 weeks	None	Week 6	79% (19/24)	50% (12/24)
Uip et al. (1998)	Adults with HIV	22	All intensity	Roxithromycin	300 mg BID for 4 weeks	None	Week 6	27% (6/23)	68% (15/22)
Woolf et al. (1987)	24 yo with HIV	1	Severe diarrhea	Spiramycin	1 g TID for 7 days	None	N/A	Relapsed	None
Wittenberg et al. (1989)	<1 yo immune-compromised infants	39	N/A	Spiramycin	75 mg/kg/d For 5 days	Placebo	Regularly until discharged	N/A	38% (8/21) vs. 33% (6/18)^b^

^a^*P* < 0.05.

^b^Not statistically significant.

a Either abatement of diarrhea or reduced stool frequency or volume.

b Either complete eradication or reduction of oocyst number.

Another macrolide antibiotic, roxithromycin, has also been proposed as one of the therapeutic options for human cryptosporidiosis. However, both published trials in HIV individuals showed unconvincing results, demonstrating a wide range of cure rates: 27–79% for clinical improvement and 50%–68% for parasite clearance (Sprinz et al., 1998; Uip et al., 1998). Spiramycin, in general did not provide clinical benefits in both immunocompetent and immunocompromised children when compared to placebo (Sáez-Llorens et al., 1989; Wittenberg et al., 1989). Conversely, the use of acetylated spiramycin in asymptomatic individuals showed a 92% oocyst reduction rate (Huang et al., 2015). Despite varying results in *Cryptosporidium* oocyst reduction, using azithromycin, erythromycin, and roxithromycin yielded desirable outcomes as abatement in diarrhea episodes (Table 4). Other macrolides, namely clarithromycin, has been studied as the chemoprophylaxis for intestinal cryptosporidiosis in individuals with HIV (Jordan, 1996). Nevertheless, clinicians must bear in mind that HIV-infected individuals with deficient CD4 levels might have been infected with other opportunistic infections, including infection caused by *Mycobacterium avium* complex, as resistance strains to azithromycin and clarithromycin have been reported (Matteelli et al., 1998); hence complicate treatment for the infection. Reported side effects on the use of macrolides include nausea, vomiting, and abdominal pain (Connolly et al., 1988; Dionisio et al., 1998; Uip et al., 1998), as well as elevated liver transaminases (Sprinz et al., 1998). Despite the seemingly well performance of macrolides in treating human cryptosporidiosis, results of these controlled trials also showed relapse of diarrhea episodes and minimum parasite clearance. Abatement of diarrhea in the study subjects might have been due to treated unrecognized co-infections causing diarrhea and not the cryptosporidiosis itself, as co-infections are common and attributing diarrhea to only single pathogen is difficult (Kotloff et al., 2013). This should emphasize the ineffectivity of macrolides as the drug of choice for intestinal cryptosporidiosis.

### 3.4 Somatostatin analogue

Somatostatin, or the growth hormone-inhibiting hormone (GHIH), has shown inhibitory effects on gastrointestinal hormones and immunomodulatory actions, particularly in the jejunum, hence its use in HIV patients with secretory diarrhea refractory to other forms of therapy (Bai et al., 2018; Katz et al., 1988). Subcutaneous injection of 50 mcg to 500 mcg of octreotide for over 2 weeks resulted in 33%–100% of resolution of diarrhea in HIV individuals with cryptosporidiosis (Cello et al., 1991; Liberti et al., 1992; Moroni et al., 1993; Romeu et al., 1991). However, none of these studies showed a decreased number of *Cryptosporidium* oocysts excreted in stools. Similarly, another study using vapreotide for 14 days resulted in low clinical response and no parasite clearance (Girard et al., 1992). As the drug must be administered through an injection, local pain has been reported following the drug administration, along with nausea and abdominal pain (Cello et al., 1991; Girard et al., 1992). Drug-induced cholecystitis and pancreatitis associated with the use of octreotide has also been mentioned elsewhere (Romeu et al., 1991). According to the published reviewed data, the use of somatostatin analogues in intestinal cryptosporidiosis is limited to HIV-infected adults to relieve the possible debilitating symptoms of diarrhea in this population, yet does not provide notable results in parasite clearance.

### 3.5 Bovine colostrum and immunoglobulins

As it is rich in immunoglobulins, the hyperimmune bovine colostrum (HBC) derived from cow vaccination during gestation has been widely studied for its potential therapeutic actions in gastrointestinal infections (Steele et al., 2013). Earlier studies found that in *Cryptosporidium*-infected calves fed with HBC showed significantly fewer diarrheic episodes along with a shorter duration of oocyst shedding (Fayer et al., 1989; Perryman et al., 1999). The HBC has also been studied as the treatment or prevention of diarrhea caused by rotavirus, *Shigella* spp., enterotoxigenic *E. coli*, and *Clostridium difficile* (Steele et al., 2013). Current review addressed several studies on the use of HBC and bovine leukocyte extract (BLE) in the treatment of human cryptosporidiosis in small groups of individuals living with HIV (Table 5). Both HBC and BLE showed over 60% rate of clinical improvement with varying degrees of parasite clearance (Florén et al., 2006; Greenberg and Cello, 1996; McMeeking et al., 1990; Nord et al., 1990; Plettenberg et al., 1993; Rump et al., 1992). However, these studies included only small number of patients in either chronic cryptosporidiosis or severe diarrhea and a notable number of subjects lost to follow-up; therefore careful interpretation of the published results. Additionally, gastrointestinal upset associated with administration of HBC has been reported in some studies (Nord et al., 1990; Plettenberg et al., 1993).

**Table 5:** Studies on the use of other treatment modalities.

Study	Population	Size	Disease severity	Drug(s)	Regimen	Comparison	Follow up period	Clinical improvement^a^	Parasite clearance^b^
Nord et al. (1990)	Individuals with HIV	5	Chronic cryptosporidiosis	Bovine colostrum (immune)	30 mg/ml For 10 days	Bovine colostrum (non-immune) 20 ml/h For 10 days	Daily	67% (2/3) vs. 0	67% (2/3) vs. 0
Greenberg and Cello (1996)	≥18 yo with HIV (CD4 <200 cells/uL)	20	Severe presentation	Bovine colostrum	10 g powder QID for 21 days	Bovine colostrum 10 g capsule QID for 21 days	Weeks 3 and 7	92% (11/12) vs. 0	35% (7/20) in total
Florén et al. (2006)	≥18 yo with HIV (CD4 <200 cells/uL)	20	Severe presentation, dehydration	Bovine colostrum (ColoPlus)	50 g BID for 4 weeks	None	Weeks 1, 5, 7	100%	N/A
Rump et al. (1992)	1-54 yo with HIV and other immune deficiencies	7	All intensity	Bovine colostrum (Lactobin)	10 g OD for 10 days	None	Days 5, 10 20	71% (5/7)	71% (5/7)
Plettenberg et al. (1993)	26-58 yo with HIV (CD4 <100 cells/uL)	7	Chronic cryptosporidiosis	Bovine colostrum (Lactobin)	10 g OD for 10 days	None	Days 5, 10 20	71% (5/7)	N/A
McMeeking et al. (1990)	31-46 yo with HIV	14	Chronic cryptosporidiosis	Bovine leukocyte extract (BLE) (immune)	5 IU Weekly for 8 weeks	BLE (non-immune) 5 IU weekly for 8 weeks	Week 8	86% (6/7) vs. 14% (1/7)^a^	N/A
Liberti et al. (1992)	37-70 yo with HIV (CD4 <100 cells/uL)	4	Severe diarrhea and dehydration	Octreotide	50 mcg up to 500 mcg s.c. TID for 21 days	None	Day 21	100%	None
Moroni et al. (1993)	21-60 yo with HIV (CD4 <100 cells/uL)	13	All intensity	Octreotide	100 mcg s.c. TID for 2 weeks with increasing dose	None	Week 4	61% (8/13)	N/A
Cello et al. (1991)	18-60 yo with HIV	15	Chronic cryptosporidiosis with severe diarrhea	Octreotide (Sandostatin)	50 mcg up to 500 mcg s.c. TID for 2 weeks with increasing dose	None	Days 14, 21 and 28	33% (5/15)	N/A
Romeu et al. (1991)	22-48 yo with HIV	18	All intensity	Octreotide (Sandostatin)	150 to 1500 mcg/day s.c. For 4 weeks	None	Week 4	72% (13/18)	N/A
Girard et al. (1992)	≥18 yo with HIV	21	All intensity	Vapreotide	400-500 mcg s.c. 2-6 times daily for 14 days	None	Day 14	38% (8/21)	None
Harris et al. (1994)	21-59 yo with HIV (CD4 <100 cells/uL)	14	All intensity	Letrazuril	50 mg OD for 6 weeks	None	Week 10	50% (7/14)	50% (7/14)
Loeb et al. (1995)	27-57 yo with HIV (CD4 <150 cells/uL)	35	All intensity	Letrazuril	50 mg OD as long as the patient responded	None	After day 7	66% (23/35)	40% (10/25)
Amenta et al. (1999)	12-54 yo with HIV	10	All intensity	Rifaximin	600 mg TID for 14 days	None	Day 14	100%	100%
Zulu et al. (2002)	≥18 yo with HIV (CD4 <200 cells/uL)	4	Chronic cryptosporidiosis	Albendazole	800 mg BID for 14 days	None	Weeks 3 and 6	60% (52/87)	100%
Sinkala et al. (2011)	≥18 yo with HIV and malnutrition	7	Chronic cryptosporidiosis	Miltefosine	2.5 mg/kg For 14 days	None	Week 4	29% (2/7)	None
Tam et al. (2020)	18-65 yo with advanced HIV	20	Chronic cryptosporidiosis	Clofazimine	50–100 mg TID For 5 days	Placebo	Daily	Worsening	None
Sindhu et al. (2014)	6 months to 5 yo healthy children	42	All intensity	Probiotic Lactobacillus rhamnosus GG	Capsule (1x10∧10 organisms) OD for 7 days	Placebo	Week 4	35% (7/20) vs. 36% (8/22)^b^	N/A

s.c.: subcutaneous.

^a^*P* < 0.05.

^b^Not statistically significant.

a Either abatement of diarrhea or reduced stool frequency or volume.

b Either complete eradication or reduction of oocyst number.

### 3.6 Other therapeutic modalities

Although large number of studies on the treatment of human intestinal cryptosporidiosis has been performed using the drugs mentioned previously, there were published results of other treatment modalities as well (Table 5). These trials mainly were conducted as either open-label pilot studies after being demonstrated promising results on laboratory-based research on the active compound of the drugs or case reports (Amenta et al., 1999; Blanshard et al., 1997). Two studies on the use of letrazuril, a benzene acetonitrile commonly used as a chemoprophylactic drug for coccidian infections in domestic fowls, showed a poor cure rate of cryptosporidiosis (Harris et al., 1994; Loeb et al., 1995). These studies were performed in severely compromised HIV individuals, which might have been affected the overall results. Additionally, the presence of elevated alkaline phosphatases and other significant side effects halted further phase 2 trials of letrazuril (Harris et al., 1994; Loeb et al., 1995).

An open-label trial on 600 mg rifaximin administered 3 times daily for 14 days showed 100% of clinical and parasitological cure in HIV individuals with CD4 ≥200 cells/uL with no notable side effects (Amenta et al., 1999). High dose of albendazole has also shown 100% parasite eradication after administration for 14 days in small number of patients with advanced HIV infection (Zulu et al., 2002). However, albendazole treatment yielded moderate gastrointestinal side effects in the study (Table 6). Despite promising results of parasitic multiplication inhibition in *in vitro* studies, miltefosine and clofazimine did not show exceptional results on clinical improvement, and none of the patients in the trials showed *Cryptosporidium* clearance (Sinkala et al., 2011; Tam et al., 2020). Furthermore, significant side effects were reported following the administration of miltefosine: elevated liver transaminases and renal failure (Sinkala et al., 2011). Worsening diarrhea episodes were also noted in patients taking clofazimine (Tam et al., 2020). The use of probiotics in the treatment of intestinal cryptosporidiosis showed no differences in reducing diarrhea severity compared to placebo (Sindhu et al., 2014). Many of these trials were terminated due to futility.

**Table 6:** Reported side effects of various drugs used for treatment of human intestinal cryptosporidiosis.

Drugs	Side effects^a^	References
Nitazoxanide	Nausea, vomiting, abdominal pain, fatigue, drowsiness, headache, dizziness, skin rash, scleral and urine discoloration, anorexia, worsening diarrhea, dry mouth, constipation	(Ali and Kumar, 2015; Amadi et al., 2009; Rossignol, 2006; Rossignol et al., 1998, 2001, 2006)
Paromomycin	Nausea, abdominal pain, elevated alkaline phosphatases	(Bissuel et al., 1994; Fichtenbaum et al., 1993; Hewitt et al., 2000; Smith et al., 1998)
Macrolides (azithromycin, spiramycin, roxithromycin, erythromycin)	Nausea, abdominal pain, vomiting, skin rash, elevated liver transaminases, worsening diarrhea	(Connolly et al., 1988; Dionisio et al., 1998; Sprinz et al., 1998; Uip et al., 1998)
Somatostatin analogue (octrotide, vapreotide)	Local pain at injection site, abdominal pain, nausea, fever, cholecystitis and pancreatitis	(Cello et al., 1991; Girard et al., 1992; Romeu et al., 1991)
Bovine colostrum and immunoglobulin	Nausea, vomiting, abdominal pain	(Nord et al., 1990; Plettenberg et al., 1993)
Letrazuril	Skin rash, elevated alkaline phosphatases, worsening diarrhea, fever	(Harris et al., 1994; Loeb et al., 1995)
Rifaximin	None	Amenta et al. (1999)
Albendazole	Nausea, vomiting	Zulu et al. (2002)
Miltefosine	Elevated liver transaminases, renal failure, intestinal obstruction, visual acuity impairment	Sinkala et al. (2011)
Clofazimine	Worsening diarrhea, abdominal pain, nausea, vomiting, malaise, anorexia	Tam et al. (2020)
Probiotics	None	Sindhu et al. (2014)

a Written from the most to the least commonly reported side effects.

## 4 Recommendations and future directions

Current review addressed diverse therapeutic modalities that have been used in clinical trials with different results that may be implemented in various settings. Nitazoxanide is the most frequently studied drug for the treatment of human intestinal cryptosporidiosis. Based on current review, a possible recommendation on the use of nitazoxanide will be the age-adjusted dose of 100 mg–500 mg given twice daily for 3 days, preferably in immunocompetent patients of all ages (Abaza et al., 2016; Amadi et al., 2002; Hussien et al., 2013; Rossignol et al., 2001, 2006), whereas in HIV individuals nitazoxanide did not demonstrate superiority than placebo (Amadi et al., 2009; Rossignol et al., 1998). The use of 500 mg of paromomycin, 3 to 4 times daily for a minimum of 14 days showed an exceptional cure rate of cryptosporidiosis, particularly in HIV-infected adults with CD4 >200 cells/uL (Bissuel et al., 1994; Fichtenbaum et al., 1993), and not in severely compromised HIV patients (Flanigan et al., 1996; Hewitt et al., 2000; Smith et al., 1998; White et al., 1994). The efficacy of paromomycin, however, was found to be inferior to nitazoxanide (Hussien et al., 2013). According to the results of the controlled trials included in the current review, the use of macrolide antibiotics showed no effective results in both clinical and parasitological improvement. The use of HBC and BLE was limited to only reducing the severity of diarrhea in immunocompromised patients (Florén et al., 2006; Greenberg and Cello, 1996; McMeeking et al., 1990; Nord et al., 1990; Plettenberg et al., 1993; Rump et al., 1992), and should possibly be administered as a complementary therapeutic drug along with other antimicrobials to reach optimal parasite eradication. Nevertheless, other than the efficacy itself, side effects should also be considered especially when the drugs are to be administered in higher doses or in a longer therapeutic regimen. The availability of drugs must be noted, as some drugs may not be available in less-developed countries. Our review demonstrated a wide variety of therapeutic options for human intestinal cryptosporidiosis. However, the number of patients included in majority of the studies was very small, and the disease spectrum and progression in which the studies were conducted was generally limited. The degree of immune suppression in HIV individuals may also play a role in clinical manifestations and therapeutic responses, as HIV-seropositive individuals with preserved CD4 levels might have had comparable immune response with that of immunocompetent individuals. Additionally, careful interpretation of data should be taken especially on the severity of diarrhea and the number of oocysts prior to any therapeutic modalities to justify the effectiveness of the treatment. This should encourage further large prospective studies in wide range of patients, both immune-competent and -compromised, and asymptomatic individuals.

Studies on the treatment of human cryptosporidiosis are generally hindered, particularly due to lack of comprehensive understanding on its immune response and mechanisms of parasite clearance, and that animal models often yielded different results in clinical trials (Sparks et al., 2015). However, more studies on novel drug targets have been performed, these include the *Cryptosporidium* calcium-dependent protein kinases (Castellanos-Gonzalez et al., 2013; Choi et al., 2020; Murphy et al., 2010), serine and cysteine proteases (Forney et al., 1996; Ndao et al., 2013), lipid kinase (Manjunatha et al., 2017), dihydrofolate reductase (Kumar et al., 2014), nucleoside diphosphate kinase (Castellanos-Gonzalez, 2020; Castellanos-Gonzalez et al., 2019), inosine monophosphate dehydrogenase (Gorla et al., 2014; Jefferies et al., 2015), and fatty acid metabolisms (Bessoff et al., 2013; Chattopadhyay and Mahapatra, 2019). Although most of these novel drug candidates have progressed through animal studies, some of these compounds were ineffective or were potentially toxic with remarkable side effects hence should be carefully selected for further development (Wang et al., 2020). In addition to introducing effective drugs to overcome the currently limited available therapeutic options for human cryptosporidiosis, raising public health awareness on the high risk of parasite transmission in a specific population of individuals should also be set in motion for better disease management and control (Ashigbie et al., 2021).

## 5 Conclusions

Current review highlights the evidences and gaps of antiparasitic treatments for human intestinal cryptosporidiosis in the past decades. Diverse therapeutic modalities in clinical trials showed various results, among which several treatments have suggested efficacy in different population and settings. However, there is inadequate data regarding controlled trials to suggest the use of these treatment modalities. Currently available data is intended to aid the development of strategies for improving access to treatments in different clinical settings, as well as to help guide further studies on anti-*Cryptosporidium* drugs.

## Ethics approval and consent to participate

Not applicable.

## Consent for publication

Not applicable.

## Availability of data and material

Data sharing not applicable to this article as no datasets were generated or analysed during the current study.

## Funding

This review did not receive any specific funding.

## Author's contributions

AD performed article extraction. Both AD and IPS conducted manuscript writing. All authors have read and approved the final manuscript.

## Declaration of competing interest

The authors declare that they have no known competing financial interests or personal relationships that could have appeared to influence the work reported in this paper.

## List of Abbreviations

AIDS: acquired immunodeficiency syndrome
ARV: antiretroviral
BID: twice daily
BLE: bovine leukocyte extract
CD4: T-helper cells
FDA: Food and Drug Administration
GHIH: growth hormone-inhibiting hormone
HBC: hyperimmune bovine colostrum
HIV: human immunodeficiency virus
IFA: immunofluorescence antibody
OD: once daily
PCR: polymerase chain reaction
PFOR: pyruvate:ferredoxin oxireductase
QID: four times daily
RCT: randomized controlled trial
TID: three times daily
